# Case report on the efficacy of phacoemulsification combined with goniosynechialysis in the treatment of advanced primary angle-closure glaucoma complicated with cataract

**DOI:** 10.1097/MD.0000000000046787

**Published:** 2026-01-02

**Authors:** Qi Wang, Junmei Zhao

**Affiliations:** a Department of Glaucoma, Shanxi Eye Hospital, Taiyuan, China.

**Keywords:** goniolysis, intraocular pressure, Primary chronic angle-closure glaucoma

## Abstract

**Rationale:**

Primary angle-closure glaucoma (PACG) is a leading cause of irreversible blindness in China, with a prevalence of as high as 1.5% in individuals over 50 years of age. Many patients are already at an advanced stage of the disease at their first clinical presentation.

**Patient concerns:**

A retrospective analysis was performed on PACG patients who underwent PEL + GSL at Shanxi Provincial Eye Hospital from June 1, 2023, to October 1, 2023.The patient noticed a decline in their vision, a narrowing of the visual field, accompanied by discomfort such as eye distension and redness. Preoperative biometry, visual field, and (Ultrasound Biomicroscopy) UBM results were recorded, and observations were made before surgery. Visual acuity, intraocular pressure, antiglaucoma medication and complications were recorded at 1week, 1month, 3months, and 6 months.

**Diagnoses:**

All eyes had advanced PACG, with an average mean deviation of -25.243 ± 6.550 dB and a pattern standard deviation of 6.516 ± 3.042. 48 cases (56 eyes) were collected, ranging in age from 47 to 86 (66.62 ± 9.10) years, and 32 (66.66%) were female patients. The preoperative visual acuity was 0.375 ± 0.245, the average mean deviation of the visual field was −25.04 ± 5.59 dB, and the pattern standard deviation was 6.516 ± 3.042.

**Interventions:**

All patients successfully underwent cataract surgery combined with goniosynechialysis.

**Outcomes:**

The intraocular pressure after PEL + GSL dropped from 27.84 ± 11.47 mm Hg before surgery to 13.696 ± 1.69 mm Hg at the last follow-up, the visual acuity improved from 0.375 ± 0.245 before surgery to 0.529 ± 0.210, and the antiglaucoma drugs decreased from 2.52 ± 0.50 before surgery to 0.11 ± 0.37.

**Lessons:**

PEL + GSL is an effective method for the treatment of advanced PACG combined with cataract, which can significantly reduce intraocular pressure with few complications.

## 1. Introduction

Advanced primary angle-closure glaucoma (PACG) with cataract poses a significant threat to vision and remains a challenging clinical condition. For this type of patient, trabeculectomy is the traditional treatment method. Recently, cataract surgery (phacoemulsification and intraocular lens implantation, PEI) combined with goniosynechialysis (GSL) (PEI + GSL) has been widely adopted for PACG management.^[1–5]^ However, no studies have analyzed its efficacy in advanced-stage glaucoma patients.^[6–9]^ In this study, we performed PEI + GSL in patients with advanced PACG and evaluated the clinical outcomes.

We retrospectively analyzed 48 patients who underwent a combined surgical procedure. Preoperative evaluations included comprehensive eye examinations, such as intraocular pressure (IOP) measurement, visual acuity testing, and gonioscopic assessment. The surgeries were conducted with strict adherence to standardized protocols. Postoperatively, the patients were systematically followed up, during which IOP, visual acuity, and changes in anterior chamber angle width were regularly monitored. The results showed remarkable improvements.

In conclusion, phacoemulsification combined with goniolysis offers a safe and effective treatment option for advanced PACG with cataracts, providing valuable guidance for clinical practice.

## 2. Methods

All surgeries were performed by an experienced physician under topical or peribulbar anesthesia. A 3.0 mm corneal incision was made. Conventional phacoemulsification surgery was performed and an intraocular lens was implanted. The surgical microscope was tilted downward by 30 to 40 degrees towards the surgeon to achieve maximum visualization under the gonioscope. The peripheral anterior synechiae were gently separated by pressing a nucleus chopper through the main incision and side incision, and the anterior chamber angle was opened over 270 °. Viscoelastic agent and hemorrhage in the anterior chamber were aspirated and cleaned. After the operation, antibiotic and corticosteroid eye drops (1% prednisolone acetate) were instilled for 7 days. The dosage of corticosteroid eye drops was gradually reduced according to the patient’s condition.

### 2.1. Subject

All patients underwent examinations, including visual acuity measurements, intraocular pressure measurements using a Goldmann applanation tonometer (AT900; Haag-Streit), slit-lamp microscope examination (BQ-900; Haag-Streit), fundus examination (with a 90 D condensing lens, Ocular), gonioscopy, ultrasound biomicroscopy (UBM) (sw-3200L), corneal endothelial cell counting (SP.3000P; TOPCON Corporation), Humphrey visual field test (Mark III; Carl Zeiss Meditec), and examination with IOL-Master 700 (Carl Zeiss Meditec).

The patient’s age, sex, eye involvement, visual acuity and intraocular pressure before and after the operation, medications for glaucoma treatment, and biometric records included axial length (AL), anterior chamber depth (ACD), lens thickness (LT), corneal endothelial cell count before the operation, mean deviation (MD) and pattern standard deviation (PSD) of the visual field, and the diameter of the implanted intraocular lens.

The data were processed using the IBM SPSS software (version 26.0, Chicago). For continuous variables, such as age, intraocular pressure (IOP) before surgery, best-corrected visual acuity (BCVA) before surgery, ACD, LT, AL, corneal endothelial cell count, MD of the visual field, PSD of the visual field, diopter of the implanted intraocular lens, and the range of anterior chamber angle synechiae, a normality test was conducted. Continuous variables conforming to a normal distribution are described as mean ± standard deviation (±s). A 1-sample t-test was used to compare the significance of the differences in visual acuity and intraocular pressure at various time periods before and after the operation, as well as the number of antiglaucoma medications used before and after the operation. A *P* significance value less than .05 was set considered to indicate a statistically significant difference.

### 2.2. Data collection

Randomised trial. This study was a retrospective analysis of patients with primary PACG who underwent PEL combined with GSL at Shanxi Eye Hospital from June 1, 2023, to October 1, 2023. Informed consent was obtained from all patients, and they signed the informed consent forms.

Inclusion criteria: The definition of PACG^[10–12]^ is that the anterior chamber angle is closed for at least 180° and there is glaucomatous optic neuropathy; the use of medications to reduce IOP, with IOP > 21 mm Hg; glaucomatous visual field defects, such as nasal step, arcuate scotoma, and paracentral scotoma, according to the Hodapp-Parrish–Anderson criteria, for advanced PACG, MD ≤−12 dB, In the central 5-degree range, there is at least 1 point with a sensitivity ≤ 15 dB. Between the central 5-degree range and the peripheral edge of the visual field, there is at least 1 point with a sensitivity of 0 dB^[12,13]^; and patients with mild or moderate cataract or those who require lens extraction. Patients with cataracts, visual acuity <0.5, or abnormal zonular fibers. Exclusion Criteria: Patients with secondary angle-closure glaucoma and those with a history of previous laser treatment or surgery were excluded.

## 3. Statistical analysis

### 3.1. General information

A total of 48 cases (56 eyes) were included in this study (Table 1). Data are presented as n (%) or the mean ± standard deviation.

**Table 1:** General information of patients with advanced PACG (mean ± standard deviation) or (%).

Items	Study eyes
Number of cases (eyes)	48 cases (56 eyes)
Male/female	16 (33.33%)/32 (66.66%)
Age	47-86 (66.62 ± 9.10)
IOP before surgery (mm Hg)	12-60 (27.839 ± 11.47)
BCVA before Surgery (logMAR)	0.375 ± 0.245
ACD (mm)	2.217 ± 0.355
LT (mm)	5.012 ± 0.405
AL (mm)	22.297 ± 1.113
Corneal endothelial cell count (cells/mm^2^)	2148.340 ± 450.88
MD (dB)	−25.243 ± 6.550
PSD (dB)	6.516 ± 3.042
Diopter of the implanted intraocular lens	23.580 ± 3.149
Range of Anterior Chamber Angle Synechiae 360°/>270°/>180°	27 (48.21%)/15 (26.79%)/14 (25%)

ACD = anterior chamber depth, AL = axial length, BCVA = best-corrected visual acuity, IOP = intraocular pressure, logMAR = logarithm of the minimum angle of resolution, LT = lens thickness, MD = mean deviation of visual field, PSD = pattern standard deviation of visual field.

### 3.2. Comparison of visual acuity, intraocular pressure, and the number of antiglaucoma medications used before and after the operation

After patients with advanced PACG were treated with PEL + GSL, the mean value of BCVA 1 week after the operation was close to that before the operation, and there was no statistically significant difference (*P* = .746). When comparing the mean values of BCVA 1 month after the operation, BCVA 3 months after the operation, and BCVA 6 months after the operation with BCVA before the operation, there were statistically significant differences (*P* < .05), and the magnitudes of the differences were moderate (Cohen d = 0.304, 0.607, and 0.758) (Table 2).

**Table 2:** Comparison of BCVA before and after the operation (mean ± standard deviation).

	BCVA before the operation	BCVA 1 wk after the operation	BCVA 1 mo after the operation	BCVA 3 mo after the operation	BCVA 6 mo after the operation
BCVA (logMAR)	0.375 ± 0.245	0.380 ± 0.238	0.439 ± 0.226	0.506 ± 0.224	0.529 ± 0.210
*P*	–	.746	.027	.001	.001
Cohen d 值	–	0.044	0.304	0.607	0.758

BCVA = best-corrected visual acuity, logMAR = logarithm of the minimum angle of resolution.

After patients with advanced PACG received PEL + GSL treatment, the mean values of IOP values at 1 day, 1 week, 1 month, 3 months, and 6 months after the operation showed statistically significant differences compared with the IOP before the operation (*P* < .05), and the magnitudes of the differences were large (Cohen d = 1.728, 4.089, 3.93, 5.636, 8.349) (Table 3).

**Table 3:** Comparison of IOP before and after the operation (mean ± standard deviation).

	IOP before the operation	IOP 1 d after the operation	IOP 1 wk after the operation	IOP 1 mo after the operation	IOP 3 mo after the operation	IOP 6 mo after the operation
IOP (mm Hg)	27.84** ± **11.47	17.268** ± **6.12	15.375** ± **3.05	15.232** ± **3.21	13.821** ± **2.49	13.696** ± **1.69
*P*	–	.001	.001	.001	.001	.001
Cohen d 值	–	1.728	4.089	3.93	5.636	8.349

IOP = intraocular pressure.

There was a statistically significant difference (*P* < .05) and a large magnitude of difference (Cohen d = 6.597) in the number of antiglaucoma medications used before and after the treatment of PEL + GSL in patients with advanced PACG (Table 4).

**Table 4:** The number of antiglaucoma medications used before and after the operation.

	Medications used before the operation	Medications used after the operation
Number (vials)	2.52 ± 0.50	0.11 ± 0.37
*P*	–	.01
Cohen d 值	–	6.597

### 3.3. Complications

After the operation, 6 eyes (10.71%) had anterior chamber hemorrhage, which was absorbed within 1 week. Anterior chamber exudation occurred in 3 eyes (5.36%) after the operation. The dosage of corticosteroids was increased, and exudation was absorbed within 2 weeks. Corneal edema was observed in 5 eyes (8.92%) after the operation, and the condition improved after 1 month. IOP increased in 6 eyes. Among them, the IOP of 4 eyes (7.14%) was controlled with 1 to 2 kinds 2 antiglaucoma medications, and malignant glaucoma occurred in 2 eyes (3.57%). The IOP was controlled after anterior vitrectomy combined with trabeculectomy. No other serious complications threatening vision were observed after the operation. Tables 5 and 6.

**Table 5:** Postoperative complications.

Postoperative Complications	Number of Eyes	Outcome
Anterior chamber hemorrhage	6 (10.71%)	Absorbed within 1 wk
Anterior chamber exudation	3 (5.36%)	Absorbed within 2 wk
Corneal edema	5 (8.92%)	Disappeared after 1 mo
Malignant glaucoma	2 (3.57%)	Anterior vitrectomy combined with trabeculectomy was performed within 1 mo
Elevated IOP	4 cases (7.14%)	Controlled by medications

IOP = intraocular pressure.

**Table 6:** Preoperative conditions of 2 cases of malignant glaucoma.

Serial number	Gender	Age	Eye	IOP before operation	Visual acuity before operation	Range of anterior chamber angle synechiae	MD/PSD	AL	LT	ACD
1	Female	68	Left	28	0.05	270°	−29.07/5.02	15.77	5.28	2.08
2	Female	47	Left	32	0.3	360°	−30.1/5.56	21.54	4.96	1.45

ACD = anterior chamber depth, AL = axial length, IOP = intraocular pressure, LT = lens thickness, MD = mean deviation of visual field, PSD = pattern standard deviation of visual field.

## 4. Discussion

In recent years, phacoemulsification for cataracts combined with goniosynechialysis has been increasingly used in the treatment of PACG.^[6–9,14]^ Rodrigues et al first reported that goniosynechialysis can increase aqueous humor outflow and reduce IOP in patients with PACG.^[9]^ Patients with advanced PACG are at high risk of surgical failure. Among the patients with advanced glaucoma in this study, more than half had received 3 or 4 types of antiglaucoma medications before the operation. For traditional filtration surgeries (trabeculectomy or trabeculectomy combined with cataract surgery), the long-term success rate depends on the presence of a filtering bleb. However, complications caused by the filtering bleb, such as cystoid macular edema, cataract, filtering bleb leakage, and filtering bleb-related endophthalmitis, increase the risk after the operation.^[15–17]^ Compared with traditional glaucoma filtration surgeries, phacotrabeculectomy with endophacoemulsification and goniosynechialysis does not involve complications related to filtering blebs and does not require long-term and close follow-up. This is especially important for patients with poor compliance and limited mobility.^[14,18]^ Some studies have reported that the success rate of phacotrabeculectomy with endophacoemulsification combined with goniosynechialysis is 80% to 95%.^[6–8]^ Wang et al evaluated the efficacy and safety of phacoemulsification with irrigation and aspiration combined with goniosynechialysis compared to trabeculectomy in the treatment of 22 eyes of patients with PACG. After a 12-month follow-up, the surgical success rate, defined as IOP < 21 mm Hg with or without local antiglaucoma medications, was 88.9%. The IOP decreased from 22.07 ± 6.62 mm Hg at baseline to 15.06 ± 3.39 mm Hg, and the number of local antiglaucoma medications decreased from 2.68 ± 1.17 to 0.78 ± 0.73 (*P* < .01).^[19]^ In this study, all the affected eyes had advanced PACG. The mean MD was -25.243 ± 6.550 dB, the PSD was 6.516 ± 3.042, and the mean cup-to-disc (C/D) ratio was 0.95. Regarding the anterior chamber angle synechiae, there were 27 cases (48.21%) with 360° synechiae, 15 cases (26.79%) with synechiae >270°, and 14 cases (25%) with synechiae >180°. After phacotrabeculectomy with endophacoemulsification combined with goniosynechialysis, the IOP decreased from 27.84 ± 11.47 mm Hg before the operation to 13.696 ± 1.69 mm Hg at the last follow-up. At the last follow-up, visual acuity increased from 0.375 ± 0.245 before the operation to 0.529 ± 0.210 at the last follow-up. The number of antiglaucoma medications decreased from 2.52 ± 0.50 before the operation to 0.11 ± 0.37 at the last follow-up. The results were similar to those mentioned above.

The mechanism of phacotrabeculectomy with endophacoemulsification combined with goniosynechialysis for the treatment of PACG is that phacoemulsification with irrigation and aspiration relieves pupillary blockage and deepens the anterior chamber. Goniosynechialysis mechanically separates posterior synechiae and reopens the areas of the anterior synechiae of the anterior chamber angle, exposing the trabecular meshwork and restoring its filtering function, which addresses the anatomical factors leading to the increase in IOP in the PACG.^[20,21]^ It enhances the outflow of aqueous humor and reduces the risk of posterior synechiae formation. Previous histological studies have shown that the pathological changes in the trabecular meshwork in PACG eyes are in a normal state compared with those in eyes with primary open-angle glaucoma.^[22]^

Goniosynechialysis is usually performed using with the assistance of a gonioscope. White et al retrospectively reported the outcomes of 17 patients with acute angle-closure glaucoma who underwent combined lens extraction and goniosynechialysis. They found that the IOP decreased significantly after surgery.^[23]^ In our study, during the 6-month follow-up period, the combination of cataract surgery and goniosynechialysis effectively controlled IOP. When corneal opacity and corneal endothelial folds affect the operation of goniosynechialysis, Some studies have shown that endoscopic goniosynechialysis (EGSL) can be used when corneal opacity and corneal endothelial folds affect goniosynechialysis. EGSL has the advantages of high magnification and a clear field of view, and anterior chamber hemorrhage and corneal edema do not affect the operation either.^[24,25]^ However, it is relatively expensive and has not been widely used in clinical practice.

As reported in early studies, phacoemulsification with irrigation and aspiration combined with goniosynechialysis surgery induces fewer complications than other surgeries.^[19]^ Previous studies have reported that the prevalence of postoperative hyphema ranges from 12.5% to 31%.^[26,27]^ In our study, hyphema occurred in 6 eyes (10.71%) and was absorbed within 1 week. Anterior chamber exudation occurred in 3 eyes (5.36%), and the condition improved after increasing the corticosteroid dosage. Corneal edema occurred in 5 eyes (8.92%) and recovered within 1 month. Elevated IOP occurred in 6 eyes (10.71%) after the operation. The IOP of 4 eyes (7.14%) was controlled with medications, and aqueous humor reflux (malignant glaucoma) occurred in 2 eyes (3.57%). The IOP was controlled after anterior vitrectomy combined with trabeculectomy. Both patients had short ALs, shallow anterior chambers, elevated preoperative IOP before the operation, and poor visual fields. Prevention of this situation in such patients before the operation will be the direction of our future research.

In conclusion, this study shows that goniosynechialysis combined with cataract surgery is an effective method for treating advanced PACG complicated by cataracts. It can significantly reduce intraocular pressure with few complications and can be widely applied in clinical practice.

## Author contributions

**Data curation:** Qi Wang.

**Writing – review & editing:** Qi Wang.

**Writing – original draft:** Junmei Zhao.
